# Cd_4_As_2_Br_3_

**DOI:** 10.1107/S160053681400395X

**Published:** 2014-02-26

**Authors:** Mohammed Kars, Thierry Roisnel, Vincent Dorcet, Allaoua Rebbah, L. Carlos Otero-Diáz

**Affiliations:** a Université Houari-Boumedienne, Faculté de Chimie, Laboratoire Sciences des Matériaux, BP 32 El-Alia Bab-Ezzouar, Algeria; b Centre de Diffractométrie X, Sciences Chimiques de Rennes, UMR 6226 CNRS Université de Rennes 1, Campus de Beaulieu, Avenue du Général Leclerc, France; c Departomento Inorgánica, Facultad C.C. Químicas, Universidad Complutense, 28040 Madrid, Spain

## Abstract

Single crystals of Cd_4_As_2_Br_3_ (tetra­cadmium biarsenide tri­bromide) were grown by a chemical transport reaction. The structure is isotypic with the members of the cadmium and mercury pnictidohalides family with general formula *M*_4_ *A*_2_ *X*_3_ (*M* = Cd, Hg; *A* = P, As, Sb; *X* = Cl, Br, I) and contains two independent As atoms on special positions with site symmetry -3 and two independent Cd atoms, of which one is on a special position with site symmetry -3. The Cd_4_As_2_Br_3_ structure consists of AsCd_4_ tetra­hedra sharing vertices with isolated As_2_Cd_6_ octa­hedra that contain As–As dumbbells in the centre of the octahedron. The Br atoms are located in the voids of this three-dimensional arrangement and bridge the different polyhedra through Cd⋯Br contacts.

## Related literature   

For structural data of the title compound extracted from X-ray powder diffraction data, see: Suchow & Stemple (1963 ▶); Rebbah & Deschanvres (1981 ▶). For classification and bond character of cadmium and mercury pnictidohalides, see: Rebbah & Rebbah (1994 ▶). For the relationship between the crystal and electronic structures based on the Zintl–Klemn concept for inter­preting and predicting the properties of these phases, see: Shevelkov & Shatruk (2001 ▶). For isotypic cadmium pnictidohalides, see: Gallay *et al.* (1975 ▶); Kassama *et al.* (1994 ▶) and for isotypic mercury pnictidohalides, see: Labbé *et al.* (1989 ▶); Shevelkov *et al.* (1996 ▶).

## Experimental   

### 

#### Crystal data  

Cd_4_As_2_Br_3_
*M* *_r_* = 839.17
Cubic, 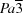

*a* = 12.625 (4) Å
*V* = 2012.4 (6) Å^3^
*Z* = 8
Mo *K*α radiation
μ = 26.70 mm^−1^
*T* = 150 K
0.21 × 0.20 × 0.17 mm

#### Data collection  

Bruker APEXII diffractometer
Absorption correction: multi-scan (*SADABS*; Bruker, 2001 ▶) *T*_min_ = 0.005, *T*_max_ = 0.011
6624 measured reflections
1485 independent reflections
1238 reflections with *I* > 2σ(*I*)
*R*_int_ = 0.088

#### Refinement  

*R*[*F* ^2^ > 2σ(*F* ^2^)] = 0.039
*wR*(*F* ^2^) = 0.092
*S* = 1.09
1485 reflections
29 parameters
Δρ_max_ = 1.93 e Å^−3^
Δρ_min_ = −1.57 e Å^−3^

### 

Data collection: *APEX2* (Bruker, 2006 ▶); cell refinement: *SAINT* (Bruker, 2006 ▶); data reduction: *SAINT*; program(s) used to solve structure: *SIR97* (Altomare *et al.*, 1999 ▶); program(s) used to refine structure: *SHELXL97* (Sheldrick, 2008 ▶); molecular graphics: *DIAMOND* (Brandenburg & Putz, 1999 ▶); software used to prepare material for publication: *WinGX* (Farrugia, 2012 ▶) and *CRYSCAL* (Roisnel, 2013 ▶).

## Supplementary Material

Crystal structure: contains datablock(s) I. DOI: 10.1107/S160053681400395X/vn2080sup1.cif

Structure factors: contains datablock(s) I. DOI: 10.1107/S160053681400395X/vn2080Isup2.hkl

CCDC reference:

Additional supporting information:  crystallographic information; 3D view; checkCIF report

## Figures and Tables

**Fig. 1.**
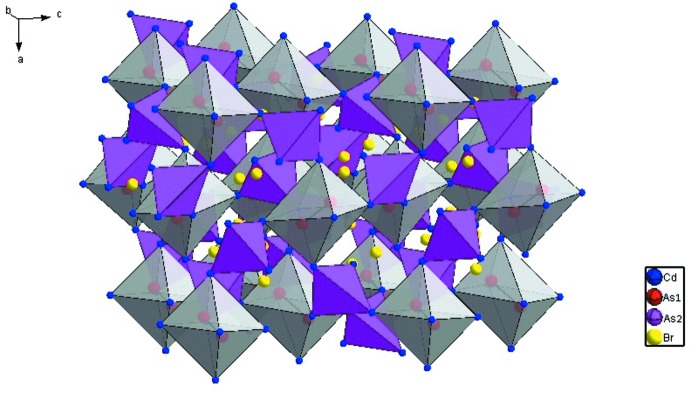
Structure de Cd4As2Br3 montrant les files octaédriques As(1)2Cd6 (en gris) reliées par les tétraèdres As(2)Cd4 (en violet).

**Fig. 2.**
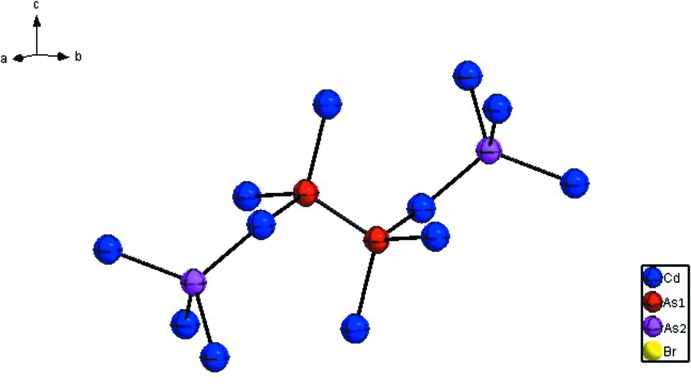
Environnement tétraèdrique distordu des atomes de As, avec un déplacement des ellipsoīdes à 95% de probabilité.

## supplementary crystallographic information

### 1. Comment

Le composé Cd_4_As_2_Br_3_ a été étudié au préalable par diffraction des rayons *X* sur poudre (Suchow & Stemple, 1963; Rebbah & Deschanvres, 1981). Ce composé appartient à la famille des composés semi-conducteurs de formule générale *M*_4_*A*_2_*X*_3_ (*M* = Cd, Hg; *A* = P, As, Sb; *X* = Cl, Br, I), connue sous le nom de halogénopnictures de cadmium et de mercure (Rebbah & Rebbah, 1994; Shevelkov & Shatruk, 2001). La principale caractéristique de ces composés polyanioniques est la présence de liaisons anion-anion (*A*—*A*). Dans le composé Cd_4_As_2_Br_3_ il existe deux types d'atomes d'arsenic ayant une coordination tétraédrique légèrement déformée. Les atomes d'arsenic de type As1 sont entourés de trois atomes Cd1 et un atome As1 de même type formant ainsi un doublet As1_2_. Les distances anion-anion As1—As1 [2.3945 (19) Å] et Cd1—As1 [2.5595 (5) Å] sont comparables à celles observées dans d'autres composés polyanioniques de la même famille: Cd_4_As_2_I_3_ [As1—As1 = 2.397 (5) Å et Cd1—As1 = 2.574 (5) Å], Gallay *et al.* (1975); Hg_4_As_2_I_3_ [As1—As1 = 2.415 (2) Å], Labbé *et al.* (1989); Hg_4_As_2_Br_3_ [As1—As1 = 2.37 (2) Å], Shevelkov *et al.* (1996). Les angles Cd1—As1—Cd1 et As1—As1—Cd1 qui valent 107.99 (2)° et 110.91 (2)° respectivement, indiquent un tétraèdre légèrement distordu. Les deux tétraèdres correspondant à une même liaison As1—As1 forment un groupement octaédrique As(1)_2_Cd_6_. Les atomes d'arsenic de type As2 ne forment pas de paire anionique et sont entourés de quatre atomes de cadmium: un atome Cd2 à 2.5655 (13) Å et trois atomes Cd1 à 2.5400 (6) Å, ces distances sont très voisines de celles autour de l'atome As1. Le tétraèdre As2Cd_4_ est aussi légèrement distordu avec des angles Cd1—As2—Cd1 et Cd1—As2—Cd2 de 118.414 (12)° et 97.29 (3)° respectivement. La charpente de cette structure est ainsi form\'ee par des octaèdres As1_2_Cd_6_ déterminant des files parallèles dans les trois directions. L'interconnexion des ces files est assurées par les tétraèdres As2Cd_4_, déterminant des cavités occupées par les atomes de brome. Ces atomes de brome assurent la cohésion de la structure par l'intermédiaire de liaisons Cd—Br en reliant d'une part deux groupements octaédriques As1_2_Cd_6_ et d'autre part un groupement octaédrique As1_2_Cd_6_ avec un groupement tétraédrique As2Cd_4_. La plus courte distance 2.7596 (7) Å est proche de celles trouvées dans Cd_4_P_2_Br_3_ [2.733 (2) Å, Kassama *et al.* 1994] ou dans Cd_4_PAsBr_3_ [2.760 (5) Å, Rebbah & Deschanvres, 1981]. Enfin l'équilibre des charges dans ce composé peut s'écrire: Cd_4_^+2^As1^-2^As2^-3^Br_3_^-^.

### 2. Experimental

Les cristaux de Cd_4_As_2_Br_3_ ont été préparés par transport en phase vapeur à partir d'un mélange stoechiométrique des éléments Cd, As et de CdBr_2_. Le mélange est broyé puis introduit dans un tube en quartz scellé; des cristaux de couleur rouge foncée sont obtenus après un chauffage à 1073 K durant une semaine.

### 3. Refinement

La structure a été déterminée par isotypie aux halogénopnictures de cadmium et de mercure de formule générale *M*_4_A_2_*X*_3_ (*M* = Cd, Hg; A = P, As, Sb; *X* = Cl, Br, I). En fin d'affinement, la carte de densité électronique obtenue est: ρ_max_ = 1.932 e Å^-3^ (localisée à 0.61 Å de C d1) et ρ_min_ = -1.571 e Å^-3^ (localisée à 1.16 Å de Br1).

### Crystal data

**Table:**

Cd_4_As_2_Br_3_	*D*_x_ = 5.54 Mg m^−^^3^
*M**_r_* = 839.17	Mo *K*α radiation, λ = 0.71073 Å
Cubic, *P**a*3	Cell parameters from 1340 reflections
Hall symbol: -P 2ac 2ab 3	θ = 2.8–34.9°
*a* = 12.625 (4) Å	µ = 26.70 mm^−^^1^
*V* = 2012.4 (6) Å^3^	*T* = 150 K
*Z* = 8	Prism, red
*F*(000) = 2904	0.21 × 0.2 × 0.17 mm

### Data collection

**Table:**

Bruker APEXII diffractometer	1238 reflections with *I* > 2σ(*I*)
Graphite monochromator	*R*_int_ = 0.088
CCD rotation images, thin slices scans	θ_max_ = 34.9°, θ_min_ = 2.8°
Absorption correction: multi-scan (*SADABS*; Bruker, 2001)	*h* = −17→15
*T*_min_ = 0.005, *T*_max_ = 0.011	*k* = −20→10
6624 measured reflections	*l* = −12→19
1485 independent reflections	

### Refinement

**Table:**

Refinement on *F*^2^	Primary atom site location: structure-invariant direct methods
Least-squares matrix: full	Secondary atom site location: difference Fourier map
*R*[*F*^2^ > 2σ(*F*^2^)] = 0.039	*w* = 1/[σ^2^(*F*_o_^2^) + (0.0052*P*)^2^ + 11.0012*P*] where *P* = (*F*_o_^2^ + 2*F*_c_^2^)/3
*wR*(*F*^2^) = 0.092	(Δ/σ)_max_ = 0.003
*S* = 1.09	Δρ_max_ = 1.93 e Å^−^^3^
1485 reflections	Δρ_min_ = −1.57 e Å^−^^3^
29 parameters	Extinction correction: *SHELXL97* (Sheldrick, 2008), Fc^*^=kFc[1+0.001xFc^2^λ^3^/sin(2θ)]^-1/4^
0 restraints	Extinction coefficient: 0.00161 (12)

### Special details

**Table:**

Geometry. All e.s.d.'s (except the e.s.d. in the dihedral angle between two l.s. planes) are estimated using the full covariance matrix. The cell e.s.d.'s are taken into account individually in the estimation of e.s.d.'s in distances, angles and torsion angles; correlations between e.s.d.'s in cell parameters are only used when they are defined by crystal symmetry. An approximate (isotropic) treatment of cell e.s.d.'s is used for estimating e.s.d.'s involving l.s. planes.
Refinement. Refinement of *F*^2^ against ALL reflections. The weighted *R*-factor *wR* and goodness of fit *S* are based on *F*^2^, conventional *R*-factors *R* are based on *F*, with *F* set to zero for negative *F*^2^. The threshold expression of *F*^2^ > σ(*F*^2^) is used only for calculating *R*-factors(gt) *etc*. and is not relevant to the choice of reflections for refinement. *R*-factors based on *F*^2^ are statistically about twice as large as those based on *F*, and *R*- factors based on ALL data will be even larger.

### Fractional atomic coordinates and isotropic or equivalent isotropic displacement parameters (Å^2^)

**Table:**

	*x*	*y*	*z*	*U*_iso_*/*U*_eq_	
Cd1	0.02627 (4)	−0.01237 (4)	0.24906 (3)	0.00853 (12)	
Cd2	0.21971 (3)	0.21971 (3)	0.21971 (3)	0.00892 (16)	
As1	0.44525 (4)	0.44525 (4)	0.44525 (4)	0.00244 (19)	
As2	0.10239 (5)	0.10239 (5)	0.10239 (5)	0.00298 (19)	
Br1	0.18751 (5)	0.43173 (4)	0.26201 (5)	0.00566 (13)	

### Atomic displacement parameters (Å^2^)

**Table:**

	*U*^11^	*U*^22^	*U*^33^	*U*^12^	*U*^13^	*U*^23^
Cd1	0.0091 (2)	0.0118 (2)	0.00468 (19)	−0.00282 (14)	0.00066 (14)	0.00389 (14)
Cd2	0.00892 (16)	0.00892 (16)	0.00892 (16)	−0.00209 (14)	−0.00209 (14)	−0.00209 (14)
As1	0.00244 (19)	0.00244 (19)	0.00244 (19)	0.00002 (17)	0.00002 (17)	0.00002 (17)
As2	0.00298 (19)	0.00298 (19)	0.00298 (19)	0.00131 (18)	0.00131 (18)	0.00131 (18)
Br1	0.0036 (2)	0.0050 (2)	0.0083 (3)	0.00048 (17)	−0.00135 (18)	−0.00099 (18)

### Geometric parameters (Å, º)

**Table:**

Cd1—As2	2.5400 (5)	As1—As1^vi^	2.3945 (19)
Cd1—As1^i^	2.5595 (5)	As1—Cd1^vii^	2.5594 (9)
Cd1—Br1^ii^	2.7933 (11)	As1—Cd1^viii^	2.5595 (5)
Cd1—Br1^iii^	2.9999 (8)	As1—Cd1^ix^	2.5595 (5)
Cd2—As2	2.5655 (13)	As2—Cd1^v^	2.5400 (6)
Cd2—Br1^iv^	2.7596 (11)	As2—Cd1^iv^	2.5400 (5)
Cd2—Br1	2.7596 (7)	Br1—Cd1^x^	2.7932 (11)
Cd2—Br1^v^	2.7596 (7)	Br1—Cd1^vii^	2.9999 (8)
			
As2—Cd1—As1^i^	140.50 (3)	Cd1^vii^—As1—Cd1^viii^	107.99 (2)
As2—Cd1—Br1^ii^	118.24 (3)	As1^vi^—As1—Cd1^ix^	110.91 (2)
As1^i^—Cd1—Br1^ii^	97.55 (2)	Cd1^vii^—As1—Cd1^ix^	107.99 (2)
As2—Cd1—Br1^iii^	106.62 (2)	Cd1^viii^—As1—Cd1^ix^	107.99 (2)
As1^i^—Cd1—Br1^iii^	91.57 (2)	Cd1^v^—As2—Cd1	118.414 (12)
Br1^ii^—Cd1—Br1^iii^	85.291 (15)	Cd1^v^—As2—Cd1^iv^	118.414 (14)
As2—Cd2—Br1^iv^	125.922 (19)	Cd1—As2—Cd1^iv^	118.416 (17)
As2—Cd2—Br1	125.923 (19)	Cd1^v^—As2—Cd2	97.29 (3)
Br1^iv^—Cd2—Br1	89.07 (3)	Cd1—As2—Cd2	97.29 (3)
As2—Cd2—Br1^v^	125.922 (17)	Cd1^iv^—As2—Cd2	97.29 (3)
Br1^iv^—Cd2—Br1^v^	89.07 (3)	Cd2—Br1—Cd1^x^	112.19 (2)
Br1—Cd2—Br1^v^	89.07 (2)	Cd2—Br1—Cd1^vii^	108.48 (2)
As1^vi^—As1—Cd1^vii^	110.91 (3)	Cd1^x^—Br1—Cd1^vii^	104.66 (2)
As1^vi^—As1—Cd1^viii^	110.91 (2)		
			
As1^i^—Cd1—As2—Cd1^v^	90.34 (7)	Br1^iv^—Cd2—As2—Cd1	129.443 (19)
Br1^ii^—Cd1—As2—Cd1^v^	−117.28 (4)	Br1—Cd2—As2—Cd1	−110.56 (2)
Br1^iii^—Cd1—As2—Cd1^v^	−23.66 (5)	Br1^v^—Cd2—As2—Cd1	9.442 (19)
As1^i^—Cd1—As2—Cd1^iv^	−114.45 (6)	Br1^iv^—Cd2—As2—Cd1^iv^	−110.56 (2)
Br1^ii^—Cd1—As2—Cd1^iv^	37.93 (6)	Br1—Cd2—As2—Cd1^iv^	9.443 (19)
Br1^iii^—Cd1—As2—Cd1^iv^	131.55 (4)	Br1^v^—Cd2—As2—Cd1^iv^	129.44 (2)
As1^i^—Cd1—As2—Cd2	−12.05 (5)	As2—Cd2—Br1—Cd1^x^	27.93 (3)
Br1^ii^—Cd1—As2—Cd2	140.33 (2)	Br1^iv^—Cd2—Br1—Cd1^x^	163.39 (3)
Br1^iii^—Cd1—As2—Cd2	−126.06 (2)	Br1^v^—Cd2—Br1—Cd1^x^	−107.53 (3)
Br1^iv^—Cd2—As2—Cd1^v^	9.443 (19)	As2—Cd2—Br1—Cd1^vii^	143.055 (14)
Br1—Cd2—As2—Cd1^v^	129.44 (2)	Br1^iv^—Cd2—Br1—Cd1^vii^	−81.486 (14)
Br1^v^—Cd2—As2—Cd1^v^	−110.557 (19)	Br1^v^—Cd2—Br1—Cd1^vii^	7.60 (2)

Symmetry codes: (i) −*x*+1/2, *y*−1/2, *z*; (ii) −*x*, *y*−1/2, −*z*+1/2; (iii) −*y*+1/2, *z*−1/2, *x*; (iv) *y*, *z*, *x*; (v) *z*, *x*, *y*; (vi) −*x*+1, −*y*+1, −*z*+1; (vii) *z*, −*x*+1/2, *y*+1/2; (viii) *y*+1/2, *z*, −*x*+1/2; (ix) −*x*+1/2, *y*+1/2, *z*; (x) −*x*, *y*+1/2, −*z*+1/2.
